# Effect of omega-3 fatty acid supplementation on serum lipids and vascular inflammation in patients with end-stage renal disease: a meta-analysis

**DOI:** 10.1038/srep39346

**Published:** 2016-12-23

**Authors:** Tianhua Xu, Yiting Sun, Wei Sun, Li Yao, Li Sun, Linlin Liu, Jianfei Ma, Lining Wang

**Affiliations:** 1 Department of Nephrology, The First Hospital of China Medical University, Shenyang 110001, China; 2 Department of Clinical Medicine, China Medical University, Shenyang 110001, China; 3 Department of General Surgery, Shengjing Hospital of China Medical University, Shenyang 110001, China

## Abstract

Omega-3 fatty acids (O3FAs) are associated with lower cardiovascular disease (CVD) risk in adults. However, this association in patients with end-stage renal disease (ESRD) remains controversial prompting the need for investigation into the role of O3FAs on serum lipids and vascular inflammation markers. The present meta-analysis summarized the effects of O3FA supplementation on serum lipids and vascular inflammatory markers in patients with ESRD. PubMed, EmBase, and the Cochrane Library were searched to identify randomized controlled trials (RCTs) focused on serum lipids and vascular inflammation markers in patients with ESRD. Standard mean differences (SMD) were used to measure the effect of O3FA supplementation on serum lipids and vascular inflammatory markers. The final pooled analysis included 20 RCTs involving 1,461 patients with ESRD. The results indicated that O3FA supplementation reduced TG by 0.61, LDL by 0.35 and CRP by 0.56. However, O3FA had no significant effect on TC, HDL, albumin, hemoglobin, homocysteine, DBP, glucose, lipoprotein(a), and ferritin. O3FA supplementation is associated with lower several serum lipids and vascular inflammation markers in patients with ESRD.

Patients with end stage renal disease (ESRD) are reported to have increasing mortality attributed to cardiovascular disease (CVD). The incidence of CVD in patients undergoing dialysis is about 3 to 45 times higher than in the general population, accounting for approximately 50% of deaths^1^,^2^,^3^. Previous observational studies investigated the role of omega-3 fatty acid (O3FAs) in reducing the CVD risk^4^,^5^,^6^. Although the mechanism of action is uncertain, O3FA-derived eicosanoids may affect physiological processes including calcium transport across cell membranes, angiogenesis, apoptosis, cell proliferation, and immune cell function, all of which strongly correlate with the risk of CVD^7^,^8^,^9^. Observational studies often misjudge the relationships due to lack of evidence on causality. Similarly, the effects of O3FA supplementation in patients with ESRD are limited and inconclusive.

A previous meta-analysis^10^ of RCTs has indicated that O3FA supplementation significantly lowered the serum triglyceride (TG) levels, with no significant changes in low-density lipoprotein (LDL), total cholesterol (TC), or high-density lipoprotein (HDL) levels. Furthermore, the effects of O3FA supplementation on reducing CVD risk factors in patients with ESRD were not confirmed by any RCT. Finally, the potential role of O3FA as treatment in patients with ESRD has not been investigated in the previous meta-analysis^10^.

Several RCTs^11^,^12^,^13^,^14^,^15^ have demonstrated that supplementation of O3FA may lower CVD risk factors, whereas other RCTs have revealed inconsistent results^16^,^17^,^18^,^19^,^20^,^21^,^22^,^23^,^24^,^25^,^26^,^27^,^28^,^29^. Hence, in this meta-analysis, RCTs were used to determine the role of O3FA supplementation on associated serum lipids and vascular inflammation markers in patients with ESRD, and assess the role of O3FA supplementation in specific subpopulations.

## Results

**Figure 1:**
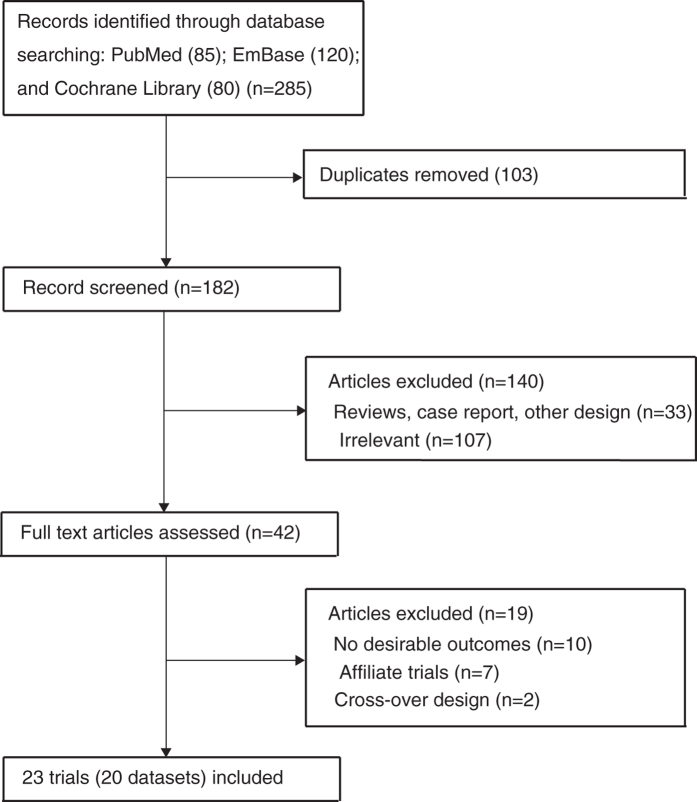
Flow diagram of the literature search and trials selection process.

The flow chart of study selection process is outlined in Fig. 1. During the initial electronic search, 285 manuscripts were identified, of which 243 were excluded including duplicates, study with other design, and unrelated studies. The full text of 42 manuscripts was retrieved, and after detailed evaluation, 23 RCTs including 20 datasets were finally selected for meta-analysis^11^,^12^,^13^,^14^,^15^,^16^,^17^,^18^,^19^,^20^,^21^,^22^,^23^,^24^,^25^,^26^,^27^,^28^,^29^,^30^,^31^,^32^,^33^. No new eligible studies were identified in manual search. The baseline characteristics of patients (n = 1,461) with ESRD are presented in Table 1. The follow-up duration of RCTs ranged from 2 to 6 months, and the number of patients enrolled in individual RCT ranged from 15 to 206. Ten trials were conducted in Asia^12^,^14^,^15^,^16^,^24^,^25^,^26^,^27^,^31^,^33^, seven in America^11^,^18^,^19^,^20^,^28^,^30^,^32^, two in Europe^17^,^23^,^29^, and one in Africa^13^. The quality of RCTs was assessed using the Jadad score^34^ (Table 1), and the scores more than 3 were regarded as high quality trials. Two trials had a score of 5^17^,^18^, six trials scored 4^12^,^16^,^19^,^31^, six trials scored 3^11^,^20^,^23^,^24^,^26^,^28^, four trials scored 2^14^,^15^,^21^,^25^, and the remaining two trials scored 1^13^,^27^.

**Figure 2:**
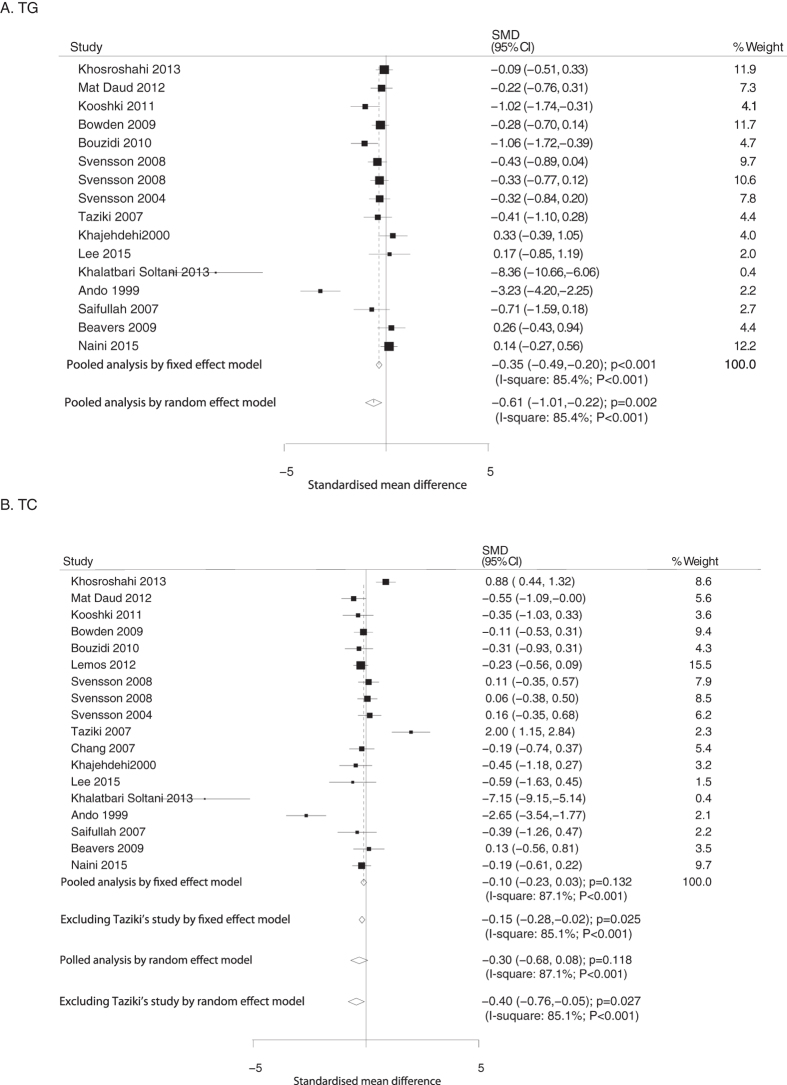
Mean changes in triglyceride (**A**) and total cholesterol (**B**) based on O3FA supplementation.

Data relating the effect of O3FA supplementation on TG were collected from 16 RCTs. Overall TG level was significantly reduced (fixed model: SMD, −0.35, 95%CI: −0.49 to −0.20, P < 0.001; random model: SMD, −0.61, 95% CI: −1.01 to −0.22, P = 0.002; Fig. 2A) in patients treated with O3FA. The extent of significant effect of O3FA was noted across all RCTs regardless of substantial heterogeneity (P < 0.001), and the conclusion was not affected after sequential exclusion of each study from pooled analyses (Table S1).

**Figure 3:**
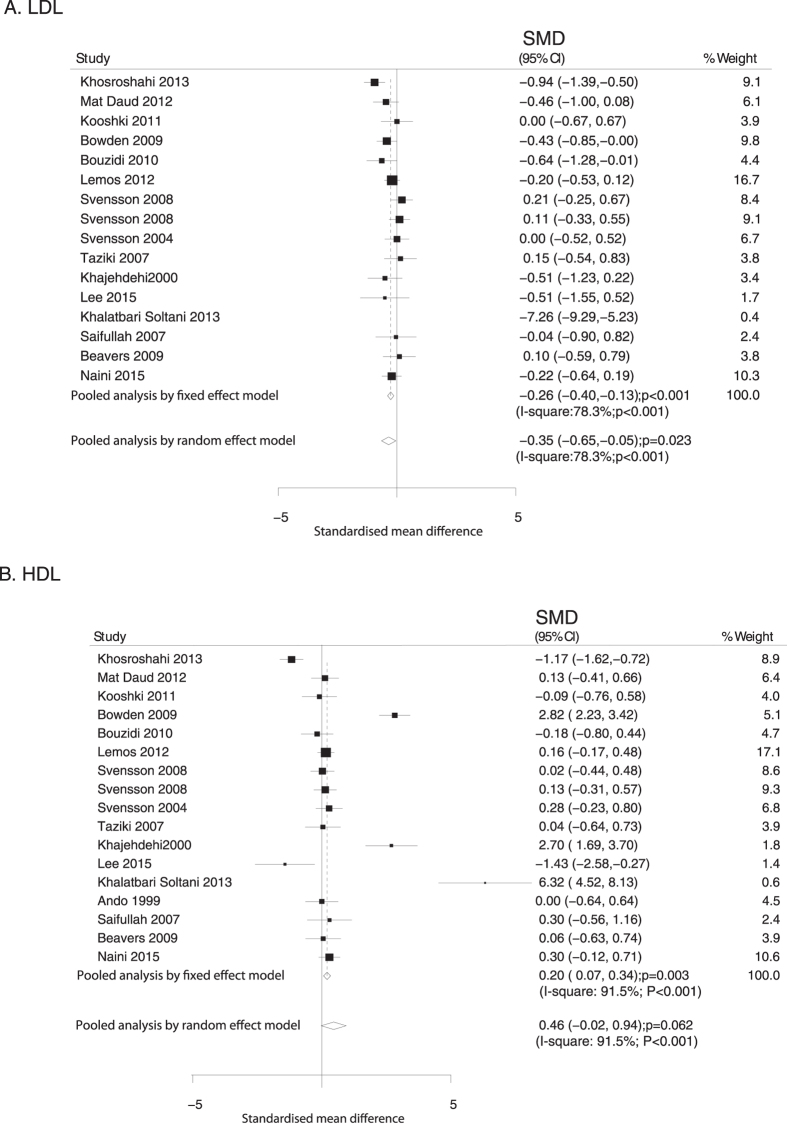
Mean changes in LDL (**A**) and HDL (**B**) based on omega-3 fatty acid supplementation.

Data relating the effect of O3FA supplementation on TC were collected from 17 RCTs. No significant difference in TC level was observed in patients treated with O3FA supplementation compared to control (fixed model: SMD, −0.10, 95%CI: −0.23 to 0.03, P = 0.132; random model: SMD, −0.30, 95%CI: −0.68 to 0.08, P = 0.118; Fig. 2B). Furthermore, substantial heterogeneity was noted across the trials (P < 0.001). The Taziki’s study^24^ was excluded from meta-analysis as younger non-diabetic patients on maintenance hemodialysis alone were enrolled in this trial. It was found that O3FA supplementation was significantly associated with reduced TC in patients with ESRD (fixed model: SMD: −0.15, 95%CI: −0.28 to −0.02, P = 0.025; random model: SMD, −0.40, 95%CI: −0.76 to −0.05, P = 0.027; Fig. 2B). Data relating the effect of O3FA supplementation on LDL and HDL were collected from 15 and 16 trials, respectively. Overall, the O3FA supplementation was associated with lower level of LDL (fixed model: SMD, −0.26, 95%CI: −0.40 to −0.13, P < 0.001; random model: SMD, −0.35, 95%CI: −0.65 to −0.05, P = 0.023; Fig. 3A). Furthermore, the pooled analysis using the fixed model revealed that O3FA supplementation was associated with a higher level of HDL (SMD: 0.20; 95%CI: 0.07 to 0.34; P = 0.003), whereas no significant difference was observed by random model (SMD: 0.46; 95%CI: −0.02 to 0.94; P = 0.062; Fig. 3B). Substantial heterogeneity was observed in the magnitude of the effect across the studies (P < 0.001), the Khosroshahi’s study was excluded from pooled analysis and we noted O3FA supplementation has little or no significant effect on LDL; furthermore, when excluding Khosroshahi or Lee’s study, O3FA supplementation significant increased the level of HDL (Table S1).

**Figure 4:**
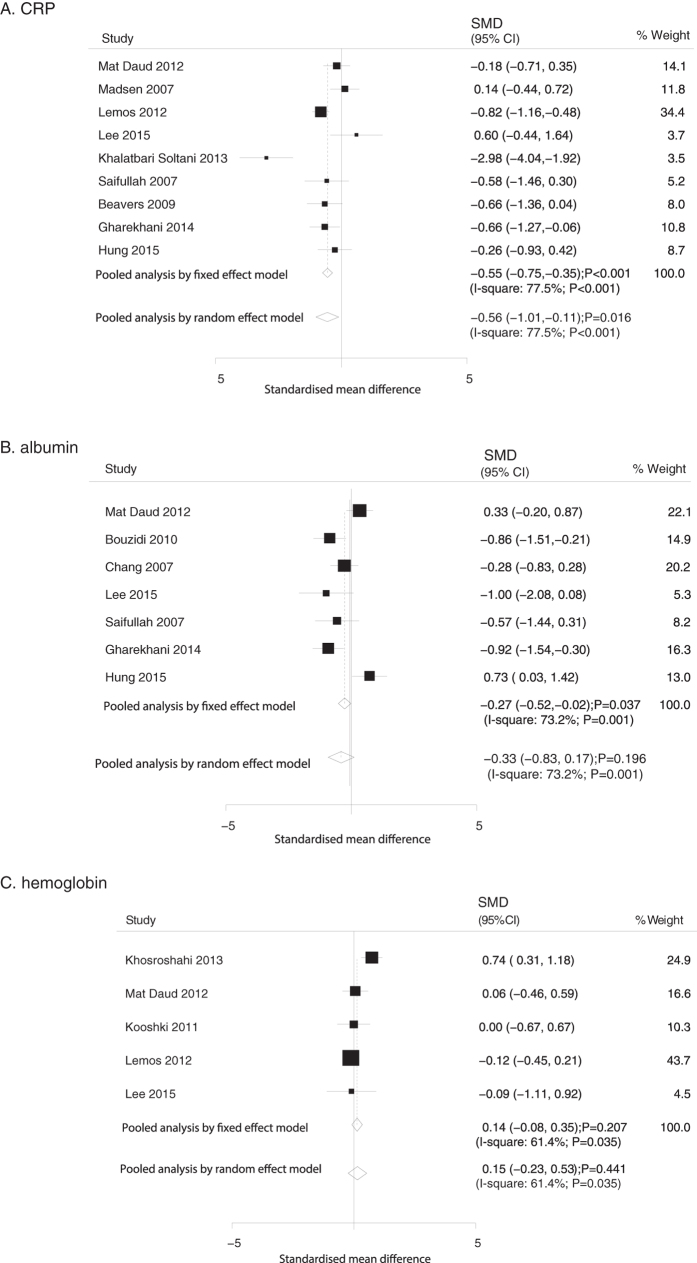
Mean changes in CRP (**A**), albumin (**B**), and hemoglobin (**C**) based on omega-3 fatty acid supplementation.

Data relating the effect of O3FA supplementation on CRP, albumin, and hemoglobin were collected from 9, 7 and 5 trials, respectively. We noted that O3FA supplementation significantly reduced the level of CRP (fixed model: SMD, −0.55, 95%CI: −0.75 to −0.35, P < 0.001; random model: SMD, −0.56, 95%CI: −1.01 to −0.11, P = 0.016; Fig. 4A). Although the summary results using fixed model indicated that O3FA supplementation was significantly associated with a reduction of albumin levels (SMD: −0.27; 95%CI: −0.52 to −0.02; P = 0.037), no significant differences were seen between O3FA and control using the random model (SMD: −0.33; 95%CI: −0.83 to 0.17; P = 0.196; Fig. 4B). In addition, no significant difference were detected in hemoglobin levels (fixed model: SMD, 0.14, 95%CI: −0.08 to 0.35, P = 0.207; random model: SMD, 0.15, 95%CI: −0.23 to 0.53, P = 0.441; Fig. 4C).

**Figure 5:**
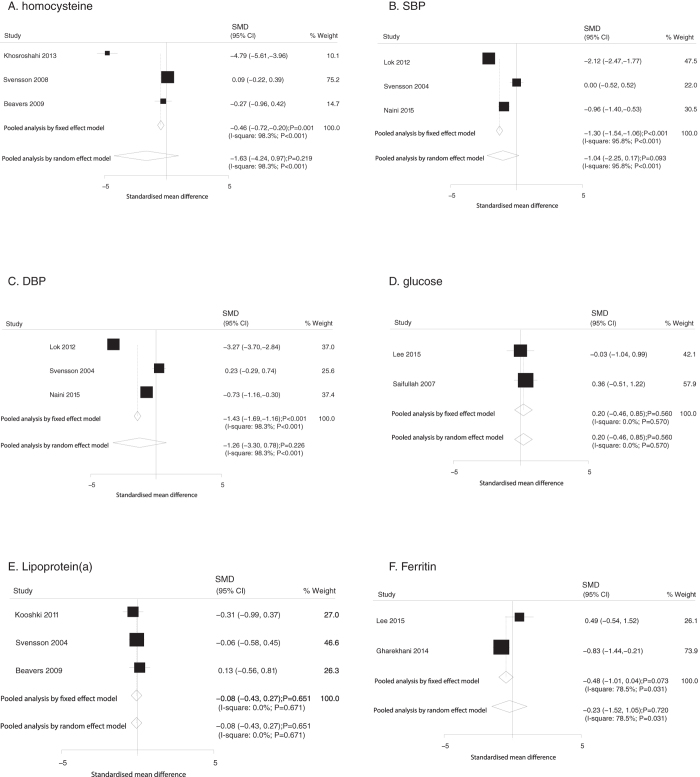
Mean changes in homocysteine (**A**), SBP (**B**), DBP (**C**), glucose (**D**), lipoprotein(a) (**E**), and ferritin (**F**) based on O3FA supplementation.

Similarly, data relating the effect of O3FA supplementation on homocysteine, SBP, DBP, glucose, lipoprotein(a), and ferritin were collected from 3, 3, 3, 2, 3, 2 trials, respectively. Overall, O3FA supplementation was not associated with altered levels of homocysteine (SMD: −1.63; 95%CI: −4.24 to 0.97; P = 0.219; Fig. 5A), SBP (SMD: −1.04; 95%CI: −2.25 to 0.17; P = 0.093; Fig. 5B), or DBP (SMD: −1.26; 95%CI: −3.30 to 0.78; P = 0.226; Fig. 5C) using the random model, whereas patients supplemented with O3FA showed significantly reduced levels of homocysteine (SMD: −0.46; 95%CI: −0.72 to −0.20; P = 0.001; Fig. 5A), SBP (SMD: −1.30; 95%CI: −1.54 to −1.06; P < 0.001; Fig. 5B), and DBP (SMD: −1.43; 95%CI: −1.69 to −1.16; P < 0.001; Fig. 5C) using the fixed model. Furthermore, O3FA supplementation had no or little effect on glucose (fixed model: SMD, 0.20; 95%CI: −0.46 to 0.85, P = 0.560; random model: SMD, 0.20; 95%CI: −0.46 to 0.85, P = 0.560; Fig. 5D), lipoprotein(a) (fixed model: SMD, −0.08, 95%CI: −0.43 to 0.27, P = 0.651; random model: SMD, −0.08, 95%CI: −0.43 to 0.27, P = 0.651; Fig. 5E), or ferritin (fixed model: SMD, −0.48, 95%CI: −1.01 to 0.04, P = 0.073; random model: SMD, −0.23, 95%CI: −1.52 to 1.05, P = 0.720; Fig. 5F) levels.

In the subgroup analysis, we stratified studies into groups to evaluate the sources of heterogeneity and explore the effect of O3FA in specific subpopulations (Table 2). First, O3FA supplementation was associated with lower TG level in multiple subsets except that the duration of follow-up was greater than 3 months. Second, O3FA supplementation significantly reduced the levels of TC in patients with BMI greater than 25.0. Third, O3FA supplementation was not associated with altered levels of LDL if the study was published before 2010, and the patients were residents of other countries, with a mean age greater than 60 years, BMI less than 25.0, and the trial showed a high quality. Forth, O3FA supplementation was associated with increased level of HDL if patients’ BMI was greater than 25.0. In addition, publication year, country, and dose of O3FA contributed significant heterogeneity between subgroups to LDL and HDL; Age and follow-up duration acted as the source of heterogeneity between subgroup for the effects of HDL; Significant heterogeneity between subgroups for TG was based on BMI; Study quality produced significant heterogeneity between subgroups for TC (Table 2).

**Figure 6:**
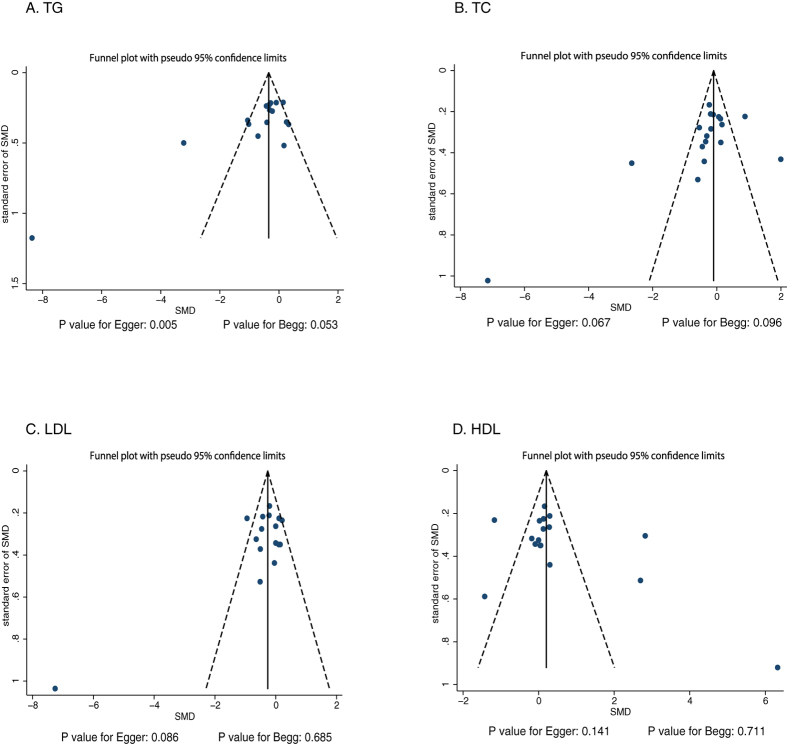
Funnel plot for TG, TC, LDL, and HDL.

Review of the funnel plots could not rule out the potential for publication bias for TG, TC, LDL, and HDL (Fig. 6). The Egger and Begg tests revealed no publication bias for TC (P values: 0.067 and 0.096, respectively), LDL (P values: 0.086 and 0.685, respectively), and HDL (P values: 0.141 and 0.711, respectively). Although the Begg test showed no evidence of publication bias for TG (P value: 0.053), the Egger test showed potential evidence of publication bias for TG (P value: 0.005). The conclusions were not changed after adjustment for publication bias by using the trim and fill method.

## Discussion

In the present meta-analysis, the effects of O3FA supplementation on serum lipids and vascular inflammation markers such as TG, TC, LDL, HDL, CRP, albumin, hemoglobin, homocysteine, SBP, DBP, glucose, lipoprotein(a) and ferritin in patients with ESRD were investigated. In this comprehensive systemic review, 1,461 patients with ESRD in 20 RCTs were included. In the absence of statistical heterogeneity (I^2^ < 50%), the fixed-effect model was used, otherwise the random effects model was applied. Therefore, the meta-analysis indicated that O3FA supplementation significantly reduced TG, LDL and CRP levels, whereas no significant effect was found in TC, HDL, albumin, hemoglobin, homocysteine, SBP, DBP, glucose, lipoprotein(a), and ferritin levels using random models. According to sensitivity analysis, O3FA supplementation was beneficial in regulating TC. Subgroup analyses revealed that O3FA supplementation significantly reduced the TG and LDL levels in several subpopulations. Furthermore, O3FA supplementation was associated with a reduced TC, LDL and increased HDL levels in patients with BMI greater than 25.0.

The methodological evaluation of each included study was limited by randomization, blinding, allocation concealment, withdrawals and dropouts, and use of intention-to-treat analysis. Our meta-analysis of RCTs provides clear randomization, blinding, and allocation concealment. Although most trials reported withdrawals and dropouts, and use of intention-to-treat analysis, the other forms of bias contributed to heterogeneity in every study. Ultimately, considering the unsatisfactory quality of included studies, we critically analyzed our recommendations for the treatment of patients with ESRD.

A previous meta-analysis^10^ suggested that O3FA significantly lowered the serum TG levels. However, there was no significant effect on LDL, TC, and HDL. The inherent limitations of the previous review were as follows several important factors such as CRP, homocysteine, SBP, DBP, glucose, albumin, hemoglobin, lipoprotein(a), and ferritin were not summarized, and the study failed to reveal the effect of O3FA supplementation on serum lipids and vascular inflammation markers in several specific subpopulations. Furthermore, a previous meta-analysis^35^,^36^ evaluated the effect of O3FA supplementation on the risk of major cardiovascular events. The effects of treatment on serum lipids and vascular inflammation markers remain unclear. Hence, a comprehensive systematic review and meta-analysis was undertaken to assess the effect of O3FA supplementation on serum lipids and vascular inflammation markers in patients with ESRD. Further subgroup analyses were performed to evaluate the effect of O3FA in subpopulations. In the present meta-analysis, the pooled SMD was <0 for TG, TC, LDL, CRP, albumin, homocysteine, SBP, DBP, lipoprotein(a) and ferritin whereas it was >0 for HDL hemoglobin, and glucose, which reflected a potential protective effect of O3FA. However, these trends were not obvious and require further validation.

Several RCTs included in this systemic review have reported inconsistent results. Many RCTs have reported that there is no significant difference between O3FAs and control on major CVD risk factors. Kooshki *et al*.^12^ conducted a RCT including 34 hemodialysis patients and found that marine O3FA supplementation could reduce serum TG, whereas it could not affect other serum lipids, lipoprotein, and hematologic factors among hemodialysis patients. Furthermore, Bouzidi *et al*.^13^ study indicated that O3FA supplementation could improve hypertriglyceridemia and oxidative stress in patients with chronic renal failure, which might lead to a decreased rate of cardiovascular complications. Khalatbari Soltani *et al*.^14^ study indicated that flax seed consumption could improve lipid abnormalities and reduce systemic inflammation in hemodialysis patients. Ando *et al*.^15^ concluded that eicosapentanoic acid administration was an effective and safe treatment to decrease plasma remnant lipoproteins. Khosroshahi *et al*.^16^ study indicated that O3FA supplementation could significantly reduce the serum homocysteine level; however, in contrast, high serum TC levels also reported. Similarly, Taziki *et al*.^24^ pointed out that O3FA was associated with higher TC levels. Khosroshahi *et al*.^16^ and Bowden *et al*.^18^ suggested that O3FA significantly reduced the LDL, whereas other trials reported no significant difference^13^,^14^. The reason could be that the sample population size was smaller than expected, and these trials were designed to evaluate other lipid factors as primary end point. Hence, clinically significant differences in LDL were not found. Similarly, Khajehdehi *et al*.^25^ and Lee *et al*.^27^ reported inconsistent results relevant to HDL when compared to other studies^14^,^16^,^18^. Some RCTs suggested that O3FA supplementation was associated with elevated HDL level^14^,^18^,^25^. On the contrary, other two trials reported that the O3FA supplementation was related with reduced HDL level^16^,^27^. The reason might be that the enrolled patients had significant heterogeneity in disease status. Moreover, two trials reported that O3FA supplementation resulted in significant reduction of homocysteine levels^16^,^21^. Lok *et al*.^19^ study suggested that O3FA was correlated with lower DBP and SBP. It was also found that O3FA supplementation could reduce the risk of intravascular clots, indicating its potential on reducing the risk of CVD.

The subgroup analysis indicated that O3FA supplementation had a significant relationship in reducing TG among multiple subsets. Notably, O3FA had no significant effect on LDL if the study published before 2010, the patients were residents of other countries, with a mean age greater than 60 years, BMI less than 25.0, and the study was a high-quality trial probably because of fewer number of trials included in these subsets. In addition, it was found that O3FA played a different role on serum lipids in different follow-up subsets. The possible reason could be that patients had different platelet count, alkaline phosphatase, serum sodium and potassium, and total iron binding capacity affected the treatment effects^37^. Another important reason could be that long-term O3FA supplementation might result in reduction of platelet activity and elimination of free radicals^38^. Finally, O3FA was associated with lower levels of TC and LDL, whereas elevated HDL level in patients with BMI was greater than 25.0. The reason for these could be that overweight participants had significant higher levels of TC and LDL than normal weight individuals, and O3FA supplementation showed reductions in BMI due to 03FA could promote fat oxidation and impair adipogenesis^39^.

A few advantages of the present meta-analysis were as follows: only RCTs were included for evaluation; the effect of O3FA supplementation in patients with ESRD was quantitatively determined using large pooled sample size; and the study provided evidence supporting the effects of O3FA supplementation on serum lipids and vascular inflammation markers such as TG, TC, LDL, HDL, CRP, albumin, hemoglobin, homocysteine, SBP, DBP, glucose, lipoprotein(a) and ferritin.

The present meta-analysis has certain limitations. First, a plenty of substantial heterogeneity among the included trials was identified in view of patients with different baseline characteristics. Second, publication bias could not be avoided when meta-analyzing published studies. Finally, more detailed relevant analysis and more comprehensive results could be restricted by conducting analysis using pooled data instead of individual data.

In summary, the findings of this meta-analysis suggested that O3FA supplementation was associated with lower serum TG, LDL, and CRP levels. Furthermore, sensitivity or subgroup analysis suggested that O3FA might play an important role on regulating TC levels. However, there was no significant difference between the effects of O3FA and control on HDL, albumin, hemoglobin, homocysteine, SBP, DBP, glucose, lipoprotein(a) and ferritin levels. Future trials should focus on specific disease status and benefits of O3FA treatment for patients with ESRD.

## Methods

### Data Sources, Search Strategy, and Selection Criteria

The present meta-analysis was performed in accordance with the Preferred Reporting Items for Systematic Reviews and Meta-Analysis protocol, 2009^40^. The RCTs designed to evaluate the influence of omega-3 supplementation on serum lipids and vascular inflammation markers were included in our study, regardless of language and publication status. Meanwhile, the impact of omega-3 supplementation on TG, TC, LDL, HDL, CRP, albumin, hemoglobin, homocysteine, systolic blood pressure (SBP), diastolic blood pressure (DBP), glucose, lipoprotein(a) and ferritin were examined. The relevant RCTs to be included in this meta-analysis were identified as follows: (1) Screening of electronic databases: PubMed, Embase, and the Cochrane Central Register of Controlled Trials were searched for studies from their inception until April 2016, using (“linolenic acid” OR “timnodonic acid” OR “ALA” OR “δ-amino linolenic acid” OR “EPA” OR “eicosapentaenoic Acid” OR “docosahexaenoic acid” OR “DHA” OR “docosahexaenoic acid” OR “omega-3 fatty acid” OR “fish oil” OR “n-3 fatty acids” OR “fatty acid” OR “omega-3” OR “α-linolenic acid” OR “eicosapentanoic acid”) AND (“kidney failure” OR “chronic renal failure” OR “dialysis” OR “hemodialysis” OR “peritoneal dialysis”) as the search terms.

(2)Other sources: ongoing (completed but not published) RCTs were identified from the metaRegister of Controlled Trials. Data pertaining to registered RCTs was obtained from the website http://clinicaltrials.gov/ (US NIH). Besides, manual searches were carried out from the reference lists within the entire relevant original and review articles in order to identify the additional eligible trials.

Two authors followed a standardized approach for conducting literature research. In case of disagreements between the two authors, mutual consensus was arrived after discussion. Since observational studies were susceptible to confounding variables and bias, the present systemic review was limited to RCTs. Subsequently, eligible studies were identified based on the following criteria: (1) patients with ESRD; (2) RCTs; (3) omega-3 fatty acid supplementation; and (4) at least one of the following variables: TG, TC, LDL, HDL, CRP, albumin, hemoglobin, homocysteine, SBP, DBP, glucose, lipoprotein(a) and ferritin. Studies were excluded if: (1) patients were diagnosed with other diseases; (2) the study was an observational study; (3) the study with inappropriate control; and (4) the mean difference was not obtained or calculated.

### Data Collection and Quality Assessment

A standardized protocol was adopted by two authors to extract all the data from included trials. The data including first author, publication year, country, sample size, mean patient’s age, sex ratio of participants, disease condition, intervention, control, outcomes, and follow-up duration were collected. In addition, data of serum lipids and vascular inflammation markers including TG, TC, LDL, HDL, CRP, albumin, hemoglobin, homocysteine, SBP, DBP, glucose, lipoprotein(a) and ferritin were collected. Simultaneously, the quality of included RCTs were assessed using Jadad score^34^, ranging from 0 to 5, on the basis of parameters including randomization, blinding, allocation concealment, withdrawals and dropouts, and use of intention-to-treat analysis.

### Statistical Analysis

The results of each RCT was considered as continuous data, and standard mean difference (SMD) and 95% confidence intervals (CIs) of each individual study were calculated from mean, standard deviation, and sample size in each group in individual RCT. Furthermore, SMD with 95%CIs were calculated for serum lipids and vascular inflammation markers, including TG, TC, LDL, HDL, CRP, albumin, hemoglobin, homocysteine, SBP, DBP, glucose, lipoprotein(a) and ferritin in patients with ESRD receiving omega-3 supplementation. The pooled SMDs of O3FA supplementation and control were compared using the fixed-effect (Mantel-Haenszel method) and random-effect models (DerSimonian-Laird method)^41^,^42^. In addition, to investigate the potential heterogeneity exist between RCTs, a subgroup analysis was performed based on the country, control, follow-up duration, and study quality. Besides, each individual trial was removed for carrying out a sensitivity analysis in the meta-analysis^43^. The heterogeneity of the treatment effects among RCTs was assessed using Cochrane Q-test; meanwhile, a *P* value of less than 0.10 was considered statistically significant^44^,^45^. P value for heterogeneity between subgroups were calculated by using Chi-square test^46^. Visual inspections of funnel plots for TG, TC, LDL, and HDL were conducted. The publication bias for TG, TC, LDL, and HDL parameters was also statistically assessed using Egger^47^ and Begg^48^ tests, and *P* values less than 0.05 was considered to have a significant publication bias. If significant publication bias was detected, trim and fill method were used to adjustment for publication bias^49^. STATA software (Version 10.0; StataCorp, Texas, United States of America) was used to perform the statistical analyses.

## Additional Information

**How to cite this article**: Xu, T. *et al*. Effect of omega-3 fatty acid supplementation on serum lipids and vascular inflammation in patients with end-stage renal disease: a meta-analysis. *Sci. Rep.* **6**, 39346; doi: 10.1038/srep39346 (2016).

**Publisher's note:** Springer Nature remains neutral with regard to jurisdictional claims in published maps and institutional affiliations.

## Supplementary Material

Supplementary Information

Supplementary Table S1

## Figures and Tables

**Table 1:** Baseline characteristics of studies included in the systematic review and meta-analysis.

Study	Country	Sample size	Mean age	Percentage male (%)	BMI (kg/m^2^)	Mean SBP (mm Hg)	History of CVD (%)	History of DM (%)	Disease status	Intervention	Control	Main outcomes	Duration of follow- up (months)	Jadad score
Khosroshahi^16^	Iran	88	50.1	72.7	NA	NA	NA	NA	Hemodialysis	Omega-3 (3 g/day)	Placebo	TG, TC, LDL, HDL, homocystein; hemoglobin	2	4
Mat Daud^11^	USA	56	58.5	50.8	27.7	NA	NA	20.0	Hemodialysis (serum albumin ≤3.9 g/dL)	Omega-3 (2.4 g/day)	Placebo	TC, HDL, LDL, TG; albumin; hemoglobin; CRP	6	3
Kooshki^12^	Iran	34	50.0	61.8	NA	NA	NA	23.5	Hemodialysis	Omega-3 (2.08 g/day)	Placebo	TG, TC, LDL, HDL; Lipoprotein(a); Hemoglobin;	2.5	4
Bowden^18^	USA	87	60.0	51.7	NA	NA	NA	59.8	ESRD treated with hemodialysis	Omega-3 (1.0 g/day)	Placebo (corn oil)	HDL, LDL, TG, TC, homocysteine	6	5
Bouzidi^13^	Algeria	40	61.0	55.0	24.2	125	NA	NA	Chronic renal failure and dyslipidemia	Omega-3 (2.1 g/day)	Counsel monitoring	TG, TC, HDL, LDL, Albumin	3	1
Lok^19^	Canada	196	62.9	50.0	NA	NA	33.7	52.6	Stage 5 chronic kidney disease	Omega-3 (4.0 g/day)	Placebo	SBP and DBP	6	4
Lemos^20^	Brazil	145	57.0	58.6	24.6	NA	NA	48.3	Hemodialysis	Omega-3 (1.0 g/day)	Placebo	TC, HDL, LDL, CRP, Hemoglobin,	4	3
Svensson and Rasmussen 2008^17^,^22^	Denmark	206	67.0	64.5	24.4	151	100	23.8	Hemodialysis and CVD	Omega-3 (1.7 g/day)	Placebo (olive oil)	TC, LDL, HDL, TG, Homocysteine	3	5
Svensson 2004^23^,^29^	Denmark	58	59.0	67.2	28.0	128	NA	NA	CRF	Omega-3 (2.4 g/day)	Placebo (olive oil)	TC, LDL, HDL, TG, DBP, SBP, Lipoprotein(a), CRP	2	3
Taziki^24^	Iran	33	53.8	33.3	24.0	NA	NA	NA	Non-diabetic patients on hemodialysis	Omega-3 (2.0 g/day)	Control group who did not receive this drug	TG, TC, HDL, and LDL	3	3
Khajehdehi^25^	Iran	60	32.4	31.0	NA	126	NA	NA	Hemodialysis	Omega-3 (1.5 g/day)	Placebo	TG, TC, LDL, and HDL	2	2
Chang^26^	Korea	50	64.5	54.0	21.2	NA	48.0	NA	ESRD patients on hemodialysis	Omega-3 (0.6 g/day)	Placebo	TC, Albumin	3	3
Lee^27^	Korea	15	62.1	33.3	NA	NA	NA	73.3	Hemodialysis	Omega-3 (2.4 g/day)	Placebo (olive oil)	TC, TG, HDL, LDL, glucose, Hemoglobin, Albumin, CRP, Ferritin	3	1
Khalatbari Soltani^14^	Iran	30	54.3	53.3	25.8	NA	NA	NA	Hemodialysis and dyslipidemia	Omega-3 (13.5 g/day)	Placebo	TC, TG, LDL, HDL, CRP	2	2
Ando^15^	Japan	38	52.5	86.5	21.0	NA	NA	42.1	Hemodialysis	Omega-3 (1.8 g/day)	Placebo	TC, TG, and HDL	3	2
Saifullah^28^	USA	23	57.7	78.3	NA	NA	NA	39.1	Hemodialysis	Omega-3 (1.3 g/day)	Placebo	LDL, TC, TG, HDL, glucose, Albumin, CRP	3	3
Beavers^21^,^30^	USA	33	60.2	42.4	NA	NA	NA	NA	ESRD	Omega-3 (1.56 g/day)	Placebo (corn-oil)	Lipoprotein(a), HDL, LDL, TG, TC, Homocysteine, CRP	6	2
Gharekhani^31^	Iran	45	56.8/57.2	52.0/60.0	NA	NA	NA	42.2	Hemodialysis patients	Omega-3 (0.9 g/day)	Placebo	CRP; Ferritin; Albumin	4	4
Hung^32^	USA	34	50.0/53.0	82.0/77.0	30.2	NA	NA	3.0	Hemodialysis patients	Omega-3 (2.9 g/day)	Placebo	CRP, Albumin	3	4
Naini^33^	Iran	90	57.7/59.4	53.0/60.0	25.3	144	NA	44.4	Continuous ambulatory peritoneal dialysis patients	Omega-3 (3.0 g/day)	Placebo	SBP, DBP, TG, TC, LDL, HDL	2	4

TG, triglyceride; TC, total cholesterol; LDL, low-density lipoprotein; HDL, high-density lipoprotein; NA, not applicable; ESRD, end stage renal disease; SBP, systolic blood pressure; DSP, diastolic blood pressure; CVD, cardiovascular disease; DM: diabetes mellitus; CKD, chronic kidney disease; CRF, chronic renal failure; CRP: C-Reactive Protein.

**Table 2:** Subgroup analysis for TG, TC, LDL, and HDL.

Outcomes	Group	SMD and 95%CI	P value	Heterogeneity (%)	P value for heterogeneity	P value for heterogeneity between subgroups
TG	**Publication year**					
	2010 or after	**−0.90 (−1.68 to −0.12)**	**0.024**	90.4	<0.001	0.641
	Before 2010	**−0.49 (−0.94 to −0.05)**	**0.031**	80.2	<0.001	
	**Country**					
	Asia	**−1.14 (−2.04 to −0.24)**	**0.013**	92.6	<0.001	0.921
	Other	**−0.36 (−0.57 to −0.15)**	**0.001**	16.8	0.298	
	**Age**					
	60 years or greater	**−0.33 (−0.63 to −0.02)**	**0.034**	41.5	0.129	0.885
	<60 years	**−0.95 (−1.60 to −0.29)**	**0.005**	90.4	<0.001	
	**BMI**					
	25 kg/m^2^ or greater	**−1.37 (−2.65 to −0.09)**	**0.035**	94.1	<0.001	**0.013**
	<25 kg/m^2^	**−1.00 (−1.76 to −0.24)**	**0.010**	87.2	<0.001	
	**Dose of O3FA**					
	3 g per day or greater	**−1.95 (−3.72 to −0.17)**	**0.031**	96.1	<0.001	0.063
	<3 g per day	**−0.51 (−0.86 to −0.16)**	**0.004**	75.3	<0.001	
	**Follow-up duration**					
	>3 months	−0.16 (−0.46 to 0.14)	0.289	0.0	0.414	0.167
	3 months or less	**−0.79 (−1.29 to −0.29)**	**0.002**	87.9	<0.001	
	**Study quality**					
	High	**−0.27 (−0.52 to −0.01)**	**0.041**	45.6	0.102	0.072
	Low	**−1.00 (−1.75 to −0.24)**	**0.010**	90.0	<0.001	
TC	**Publication year**					
	2010 or after	−0.60 (−1.24 to 0.04)	0.067	90.2	<0.001	0.444
	Before 2010	−0.11 (−0.59 to 0.37)	0.644	84.9	<0.001	
	**Country**					
	Asia	−0.71 (−1.60 to 0.18)	0.117	93.6	<0.001	0.800
	Other	−0.11 (−0.27 to 0.05)	0.177	0.0	0.554	
	**Age**					
	60 years or greater	−0.06 (−0.26 to 0.14)	0.561	0.0	0.808	0.625
	<60 years	−0.53 (−1.16 to 0.11)	0.106	92.2	<0.001	
	**BMI**					
	25 kg/m^2^ or greater	**−1.34 (−2.58 to −0.10)**	**0.034**	93.9	<0.001	0.257
	<25 kg/m^2^	−0.16 (−0.78 to 0.47)	0.621	89.7	<0.001	
	**Dose of O3FA**					
	3 g per day or greater	−1.68 (−3.66 to 0.31)	0.098	96.9	<0.001	0.071
	<3 g per day	−0.20 (−0.52 to 0.12)	0.223	78.2	<0.001	
	**Follow-up duration**					
	>3 months	−0.21 (−0.43 to 0.01)	0.057	0.0	0.454	0.209
	3 months or less	−0.39 (−0.91 to 0.13)	0.142	89.8	<0.001	
	**Study quality**					
	High	0.08 (−0.26 to 0.43)	0.635	70.3	0.005	**0.004**
	Low	−0.59 (−1.18 to 0.00)	0.051	89.7	<0.001	
LDL	**Publication year**					
	2010 or after	**−0.78 (−1.33 to −0.22)**	**0.006**	86.9	<0.001	**0.003**
	Before 2010	−0.05 (−0.24 to 0.14)	0.612	0.0	0.453	
	**Country**					
	Asia	**−0.88 (−1.64 to −0.12)**	**0.024**	88.8	<0.001	**0.019**
	Other	−0.15 (−0.34 to 0.03)	0.108	18.6	0.277	
	**Age**					
	60 years or greater	−0.15 (−0.44 to 0.15)	0.339	41.2	0.131	0.130
	<60 years	**−0.52 (−0.98 to −0.06)**	**0.028**	84.5	<0.001	
	**BMI**					
	25 kg/m^2^ or greater	**−1.36 (−2.57 to −0.15)**	**0.028**	93.6	<0.001	0.110
	<25 kg/m^2^	−0.06 (−0.33 to 0.20)	0.637	34.8	0.189	
	**Dose of O3FA**					
	3 g per day or greater	**−2.25 (−3.96 to −0.54)**	**0.010**	95.7	<0.001	**0.001**
	<3 g per day	**−0.15 (−0.30 to −0.00)**	**0.046**	1.2	0.434	
	**Follow-up duration**					
	>3 months	**−0.28 (−0.50 to −0.05)**	**0.014**	0.0	0.514	0.885
	3 months or less	**−0.44 (−0.88 to −0.01)**	**0.047**	83.5	<0.001	
	**Study quality**					
	High	−0.23 (−0.58 to 0.12)	0.206	70.7	0.004	0.787
	Low	**−0.52 (−1.02 to −0.02)**	**0.040**	82.7	<0.001	
HDL	**Publication year**					
	2010 or after	0.20 (−0.49 to 0.88)	0.575	91.4	<0.001	<**0.001**
	Before 2010	0.68 (−0.00 to 1.36)	0.051	91.1	<0.001	
	**Country**					
	Asia	0.63 (−0.33 to 1.58)	0.197	93.5	<0.001	**0.012**
	Other	0.41 (−0.12 to 0.94)	0.131	89.2	<0.001	
	**Age**					
	60 years or greater	0.28 (−0.70 to 1.26)	0.578	93.8	<0.001	**0.044**
	<60 years	0.53 (−0.03 to 1.09)	0.066	90.4	<0.001	
	**BMI**					
	25 kg/m^2^ or greater	**1.27 (0.12 to 2.41)**	**0.030**	93.0	<0.001	0.067
	<25 kg/m^2^	0.07 (−0.12 to 0.27)	0.471	0.0	0.957	
	**Dose of O3FA**					
	3 g per day or greater	1.50 (−0.62 to 3.62)	0.165	97.3	<0.001	**0.005**
	<3 g per day	0.35 (−0.10 to 0.80)	0.124	87.9	<0.001	
	**Follow-up duration**					
	>3 months	0.78 (−0.41 to 1.98)	0.200	95.4	<0.001	<**0.001**
	3 months or less	0.34 (−0.19 to 0.86)	0.206	89.1	<0.001	
	**Study quality**					
	High	0.33 (−0.62 to 1.27)	0.497	95.5	<0.001	0.714
	Low	0.52 (−0.04 to 1.07)	0.069	87.2	<0.001	

TG, triglyceride; TC, total cholesterol; LDL, low-density lipoprotein; HDL, high-density lipoprotein; CI, confidence interval; SMD, standard mean difference; BMI, body mass index; O3FA, omega-3 fatty acid.
