# Supplementary material for: Effect of omega-3 fatty acid supplementation on serum lipids and vascular inflammation in patients with end-stage renal disease: a meta-analysis
Source: Sci Rep. 2016 Dec 23;6:39346. doi: 10.1038/srep39346 (PMC5180246; doi:10.1038/srep39346)
Supplement: Supplementary Information [file srep39346-s1.doc]

**Effect of** **omega-3 fatty acids supplementation** **on serum lipids and vascular inflammation markers in patients with end-stage renal disease: a meta-analysis**

Tianhua Xu^1#^, Yiting Sun^2#^, Wei Sun^3^, Li Yao^1*^, Li Sun^1^, Linlin Liu^1^, Jianfei Ma^1^, Lining Wang^1^

^1^Department of Nephrology, The First Hospital of China Medical University, Shenyang 110001, China.

^2^Department of Clinical Medicine, China Medical University, Shenyang 110001, China.

^3^Department of General Surgery, Shengjing Hospital of China Medical University, Shenyang 110001, China

^#^These authors contributed equally to this study.

**^*^Corresponding author:**

Li Yao

Department of Nephrology, The First Hospital of China Medical University, Shenyang 110001, China.

Tel: +8618040096533

Email: [liyaosci@sina.com](mailto:liyaosci@sina.com)

| **PubMed** | **Search strategy** |
| --- | --- |
| #1 | "Fatty Acids, Omega-3"[Mesh] OR "Eicosapentaenoic Acid"[Mesh] OR "Docosahexaenoic Acids"[Mesh] OR "alpha-Linolenic Acid"[Mesh]" |
| #2 | "Omega-3" OR "Eicosapentaenoic Acid" OR "EPA" OR "Docosahexaenoic acid" OR "DHA" OR "ω-3 " OR "linolenic acid" OR "timnodonic acid" OR "alpha-Linolenic Acid" OR "ALA" OR "δ-amino linolenic acid" OR "omega-3 fatty acid" OR "α-linolenic acid" |
| #3 | #1 OR #2 |
| #4 | "Renal Insufficiency"[Mesh] OR "Kidney Failure, Chronic"[Mesh] OR "Acute Kidney Injury"[Mesh] OR "Uremia"[Mesh] |
| #5 | "renal insufficiency" OR "chronic kidney failure" OR "ESRD" OR "renal failure" OR "acute kidney injury" OR "uremia" |
| #6 | #4 OR #5 |
| #7 | #3 AND #6 |
| #8 | "Randomized Controlled Trial"[Publication Type] OR "Randomized Controlled Trials as Topic"[Mesh] OR "Controlled Clinical Trial"[Publication Type] |
| #9 | "randomized controlled trial" OR "RCT*" OR "controlled clinical trial" |
| #10 | #8 AND #9 |
| #11 | #7 AND #10 |

| **Embase** | **Search strategy** |
| --- | --- |
| **#1** | 'omega 3 fatty acid'/exp OR 'bilantin omega' OR 'conchol 36' OR 'eicosa e' OR 'eicosapen' OR 'epaisdin' OR 'epanova' OR 'fatty acids, omega 3' OR 'fatty acids, omega-3' OR 'n 3 fatty acid' OR 'n 3 polyunsaturated fatty acid' OR 'omega 3' OR 'omega 3 carboxylic acid' OR 'omega 3 carboxylic acids' OR 'omega 3 fatty acid' OR 'omega 3 feingold' OR 'omega 3 plus' OR 'omega 3 polyunsaturated fatty acid' OR 'omega forte' OR 'omega-3-carboxylic acids' OR 'omega3 polyunsaturated fatty acid' OR 'sakana' OR 'sanhelios omega 3' OR 'icosapentaenoic acid'/exp OR '5, 8, 11, 14, 17 eicosapentaenoic acid' OR '5, 8, 11, 14, 17 icosapentaenoic acid' OR '5, 8, 11, 14, 17-eicosapentaenoic acid' OR 'eicosa 5, 8, 11, 14, 17 pentaene carboxylic acid' OR 'eicosa 5, 8, 11, 14, 17 pentaenoic acid' OR 'eicosapentaenoate' OR 'eicosapentaenoic acid' OR 'eicosapentenoic acid' OR 'icosa 5, 8, 11, 14, 17 pentaenoic acid' OR 'icosapent' OR 'icosapentaenoate' OR 'icosapentaenoic acid' OR 'omega 3 eicosapentaenoic acid' OR 'unsaturated fatty acid'/exp OR 'ufa' OR 'alkenyl fatty acid' OR 'fats, unsaturated' OR 'fatty acid, unsaturated' OR 'fatty acids, unsaturated' OR 'unsaturated fat' OR 'unsaturated fatty acid' OR 'unsaturated lipid' OR 'cervonic acid'/exp OR '4, 7, 10, 13, 16, 19 docosahexaenoate' OR '4, 7, 10, 13, 16, 19 docosahexaenoic acid' OR 'cervonic acid' OR 'doconexent' OR 'docosa 4, 7, 10, 13, 16, 19 hexaenoic acid' OR 'omega 3 docosahexaenoate' OR 'omega 3 docosahexaenoic acid' |
| **#2** | 'kidney failure'/exp OR 'kidney failure' OR 'kidney insufficiency' OR 'maternal kidney failure' OR 'renal failure' OR 'renal insufficiency' OR 'terminal kidney failure' OR 'acute kidney failure'/exp OR 'chronic kidney failure'/exp OR 'end stage renal disease'/exp OR 'uremia'/exp |
| #3 | 'randomized controlled trial'/exp OR 'controlled clinical trial (topic)'/exp OR 'randomized controlled trial (topic)'/exp OR 'controlled clinical trial'/exp |
| #4 | #1 AND #2 AND #3 |

| **Cochrane** | **Search strategy** |
| --- | --- |
| #1 | [Fatty Acids, Omega-3] explode all trees |
| #2 | [Eicosapentaenoic Acid] explode all trees |
| #3 | [alpha-Linolenic Acid] explode all trees |
| #4 | [Docosahexaenoic Acids] explode all trees |
| #5 | "Omega-3" or "Eicosapentaenoic Acid" or "EPA" or "Docosahexaenoic acid" or "DHA" or "ω-3 " or "linolenic acid" or "timnodonic acid" or "alpha-Linolenic Acid" or "ALA" or "δ-amino linolenic acid" or "omega-3 fatty acid" or "α-linolenic acid" |
| #6 | #1 or #2 or #3 or #4 or #5 |
| #7 | [Renal Insufficiency] explode all trees |
| #8 | [Kidney Failure, Chronic] explode all trees |
| #9 | [Acute Kidney Injury] explode all trees |
| #10 | [Cardio-Renal Syndrome] explode all trees |
| #11 | "renal insufficiency" or "chronic kidney failure" or "ESRD" or "renal failure" or "acute kidney injury" or "uremia" |
| #12 | [Uremia] explode all trees |
| #13 | #7 or #8 or #9 or #10 or #11 or #12 |
| #14 | #6 and #13 |
