# Supplementary material for: Effect of omega-3 fatty acid supplementation on serum lipids and vascular inflammation in patients with end-stage renal disease: a meta-analysis
Source: Sci Rep. 2016 Dec 23;6:39346. doi: 10.1038/srep39346 (PMC5180246; doi:10.1038/srep39346)
Supplement: Supplementary Table S1 [file srep39346-s2.doc]

**Effect of** **omega-3 fatty acid supplementation** **on serum lipids and vascular inflammation in patients with end-stage renal disease: a meta-analysis**

Tianhua Xu^1#^, Yiting Sun^2#^, Wei Sun^3^, Li Yao^1*^, Li Sun^1^, Linlin Liu^1^, Jianfei Ma^1^, Lining Wang^1^

^1^Department of Nephrology, The First Hospital of China Medical University, Shenyang 110001, China.

^2^Department of Clinical Medicine, China Medical University, Shenyang 110001, China.

^3^Department of General Surgery, Shengjing Hospital of China Medical University, Shenyang 110001, China

^#^These authors contributed equally to this study.

**^*^Corresponding author:**

Li Yao

Department of Nephrology, The First Hospital of China Medical University, Shenyang 110001, China.

Tel: +8618040096533

Email: [liyaosci@sina.com](mailto:liyaosci@sina.com)

Table S1. Sensitivity analyses

| **Outcomes** | **Excluding study** | **SMD and 95%CI** | **P value** | **Heterogeneity (%)** | **P value for heterogeneity** |
| --- | --- | --- | --- | --- | --- |
| TG | **Khosroshahi 2013** | **-0.67 (-1.11 to -0.24)** | **0.003** | **86.2** | **<0.001** |
|  | **Mat Daud 2012** | **-0.65 (-1.08 to -0.23)** | **0.003** | **86.3** | **<0.001** |
|  | **Kooshki 2011** | **-0.59 (-1.00 to -0.17)** | **0.005** | **85.9** | **<0.001** |
|  | **Bowden 2009** | **-0.66 (-1.10 to -0.22)** | **0.003** | **86.4** | **<0.001** |
|  | **Bouzidi 2010** | **-0.58 (-1.00 to -0.17)** | **0.006** | **85.7** | **<0.001** |
|  | **Svensson 2008** | **-0.64 (-1.08 to -0.21)** | **0.004** | **86.4** | **<0.001** |
|  | **Svensson 2008** | **-0.65 (-1.09 to -0.22)** | **0.003** | **86.4** | **<0.001** |
|  | **Svensson 2004** | **-0.65 (-1.08 to -0.22)** | **0.003** | **86.4** | **<0.001** |
|  | **Taziki 2007** | **-0.64 (-1.06 to -0.21)** | **0.003** | **86.4** | **<0.001** |
|  | **Khajehdehi 2000** | **-0.68 (-1.09 to -0.26)** | **0.001** | **85.9** | **<0.001** |
|  | **Lee 2015** | **-0.66 (-1.07 to -0.25)** | **0.002** | **86.2** | **<0.001** |
|  | **Khalatbari Soltani 2013** | **-0.42 (-0.72 to -0.12)** | **0.007** | **75.0** | **<0.001** |
|  | **Ando 1999** | **-0.42 (-0.77 to -0.08)** | **0.015** | **79.7** | **<0.001** |
|  | **Saifullah 2007** | **-0.61 (-1.03 to -0.20)** | **0.004** | **86.3** | **<0.001** |
|  | **Beavers 2009** | **-0.68 (-1.09 to -0.26)** | **0.001** | **86.0** | **<0.001** |
|  | **Naini 2015** | **-0.68 (-1.11 to -0.26)** | **0.002** | **85.5** | **<0.001** |
| TC | Khosroshahi 2013 | -0.37 (-0.74 to 0.00) | 0.052 | 85.6 | <0.001 |
|  | Mat Daud 2012 | -0.29 (-0.69 to 0.11) | 0.151 | 87.6 | <0.001 |
|  | Kooshki 2011 | -0.30 (-0.70 to 0.09) | 0.134 | 87.8 | <0.001 |
|  | Bowden 2009 | -0.32 (-0.73 to 0.08) | 0.119 | 87.9 | <0.001 |
|  | Bouzidi 2010 | -0.31 (-0.70 to 0.09) | 0.131 | 87.8 | <0.001 |
|  | Lemos 2012 | -0.32 (-0.74 to 0.10) | 0.130 | 87.8 | <0.001 |
|  | Svensson 2008 | -0.34 (-0.74 to 0.07) | 0.102 | 87.8 | <0.001 |
|  | Svensson 2008 | -0.33 (-0.74 to 0.07) | 0.106 | 87.8 | <0.001 |
|  | Svensson 2004 | -0.34 (-0.74 to 0.06) | 0.098 | 87.8 | <0.001 |
|  | **Taziki 2007** | **-0.40 (-0.76 to -0.05)** | **0.027** | **85.1** | **<0.001** |
|  | Chang 2007 | -0.31 (-0.71 to 0.08) | 0.122 | 87.9 | <0.001 |
|  | Khajehdehi 2000 | -0.30 (-0.69 to 0.10) | 0.141 | 87.8 | <0.001 |
|  | Lee 2015 | -0.29 (-0.68 to 0.10) | 0.145 | 87.8 | <0.001 |
|  | Khalatbari Soltani 2013 | -0.13 (-0.44 to 0.18) | 0.414 | 81.0 | <0.001 |
|  | Ando 1999 | -0.15 (-0.49 to 0.19) | 0.380 | 83.8 | <0.001 |
|  | Saifullah 2007 | -0.30 (-0.69 to 0.09) | 0.135 | 87.8 | <0.001 |
|  | Beavers 2009 | -0.33 (-0.72 to 0.06) | 0.101 | 87.8 | <0.001 |
|  | Naini 2015 | -0.32 (-0.73 to 0.09) | 0.125 | 87.8 | <0.001 |
| LDL | Khosroshahi 2013 | -0.30 (-0.60 to 0.11) | 0.057 | 76.2 | <0.001 |
|  | **Mat Daud 2012** | **-0.35 (-0.67 to -0.02)** | **0.035** | **79.5** | **<0.001** |
|  | **Kooshki 2011** | **-0.38 (-0.70 to -0.06)** | **0.020** | **79.5** | **<0.001** |
|  | **Bowden 2009** | **-0.35 (-0.68 to -0.02)** | **0.035** | **79.5** | **<0.001** |
|  | **Bouzidi 2010** | **-0.33 (-0.65 to -0.02)** | **0.040** | **79.3** | **<0.001** |
|  | **Lemos 2012** | **-0.38 (-0.72 to -0.04)** | **0.029** | **79.7** | **<0.001** |
|  | **Svensson 2008** | **-0.40 (-0.71 to -0.08)** | **0.015** | **78.3** | **<0.001** |
|  | **Svensson 2008** | **-0.39 (-0.71 to -0.07)** | **0.017** | **78.8** | **<0.001** |
|  | **Svensson 2004** | **-0.38 (-0.71 to -0.06)** | **0.020** | **79.4** | **<0.001** |
|  | **Taziki 2007** | **-0.39 (-0.70 to -0.07)** | **0.017** | **79.3** | **<0.001** |
|  | **Khajehdehi2000** | **-0.34 (-0.66 to -0.03)** | **0.034** | **79.6** | **<0.001** |
|  | **Lee 2015** | **-0.35 (-0.66 to -0.03)** | **0.031** | **79.6** | **<0.001** |
|  | **Khalatbari Soltani 2013** | **-0.23 (-0.41 to -0.05)** | **0.013** | **39.8** | **0.056** |
|  | **Saifullah 2007** | **-0.37 (-0.69 to -0.05)** | **0.022** | **79.6** | **<0.001** |
|  | **Beavers 2009** | **-0.38 (-0.70 to -0.07)** | **0.018** | **79.4** | **<0.001** |
|  | **Naini 2015** | **-0.37 (-0.70 to -0.04)** | **0.028** | **79.7** | **<0.001** |
| HDL | **Khosroshahi 2013** | **0.56 (0.09 to 1.03)** | **0.019** | **90.0** | **<0.001** |
|  | Mat Daud 2012 | 0.49 (-0.03 to 1.01) | 0.063 | 92.1 | <0.001 |
|  | Kooshki 2011 | 0.50 (-0.01 to 1.01) | 0.054 | 92.0 | <0.001 |
|  | Bowden 2009 | 0.26 (-0.14 to 0.65) | 0.206 | 86.5 | <0.001 |
|  | Bouzidi 2010 | 0.51 (-0.00 to 1.02) | 0.051 | 92.0 | <0.001 |
|  | Lemos 2012 | 0.50 (-0.05 to 1.04) | 0.072 | 92.1 | <0.001 |
|  | Svensson 2008 | 0.50 (-0.02 to 1.02) | 0.061 | 92.0 | <0.001 |
|  | Svensson 2008 | 0.49 (-0.03 to 1.02) | 0.065 | 92.1 | <0.001 |
|  | Svensson 2004 | 0.48 (-0.04 to 1.00) | 0.069 | 92.1 | <0.001 |
|  | Taziki 2007 | 0.49 (-0.02 to 1.00) | 0.058 | 92.0 | <0.001 |
|  | Khajehdehi2000 | 0.33 (-0.14 to 0.80) | 0.171 | 90.9 | <0.001 |
|  | **Lee 2015** | **0.55 (0.07 to 1.04)** | **0.026** | **91.7** | **<0.001** |
|  | Khalatbari Soltani 2013 | 0.25 (-0.19 to 0.68) | 0.264 | 89.6 | <0.001 |
|  | Ando 1999 | 0.50 (-0.02 to 1.01) | 0.057 | 92.0 | <0.001 |
|  | Saifullah 2007 | 0.47 (-0.03 to 0.98) | 0.066 | 92.1 | <0.001 |
|  | Beavers 2009 | 0.49 (-0.02 to 1.00) | 0.059 | 92.1 | <0.001 |
|  | Naini 2015 | 0.48 (-0.04 to 1.01) | 0.073 | 92.0 | <0.001 |
